# Association Between Gut Microbiota Dysbiosis and Synovial Inflammation in Carpal Osteoarthritis of Ili Racehorses: A Preliminary Study Integrating Imaging, Synovial Fluid Analysis, and Fecal 16S rRNA Sequencing

**DOI:** 10.3390/ani16203299

**Published:** 2026-10-20

**Authors:** Jiaqi Wang, Xiaxin Liu, Mengyang Hou, Zhiheng Zhang, Zongsheng Qiu, Yuhui Ma, Jiantao Zhang, Yanan Li, Tianwen Ma

**Affiliations:** 1 Heilongjiang Provincial Key Laboratory of Pathogenic Mechanism for Animal Disease and Comparative Medicine, College of Veterinary Medicine, Northeast Agricultural University, Harbin 150036, China; 18745253011@163.com (J.W.); liuxiaxin134@163.com (X.L.); 18637350340@163.com (M.H.); qzs1077@163.com (Z.Q.); zhangjiantao@neau.edu.cn (J.Z.); 2 College of Veterinary Medicine, Inner Mongolia Agricultural University, Hohhot 010208, China; zzh449756020@163.com; 3 Zhaosu County Xiyu Horse Industry Co., Ltd., Xinjiang 835500, China; xymymayuhui1986@163.com

**Keywords:** osteoarthritis, gut microbiota, synovial fluid, carpal

## Abstract

Osteoarthritis (OA) is a leading cause of lameness and premature retirement in racehorses. Gut microbiota may contribute to OA pathogenesis through the gut–joint axis, but this relationship remains unclear in racehorses. This study focused on six OA-affected Ili racehorses and six healthy Ili racehorses from the same region; the Ili horse originated from Yili, Xinjiang, China. OA severity was assessed by clinical examination, lameness grading, radiography, arthroscopy, synovial fluid biomarker analysis, and fecal 16S rRNA gene sequencing. Associations between differential gut microbiota and synovial inflammatory markers were evaluated by Spearman correlation analysis. OA horses exhibited higher lameness scores and typical radiographic findings. Compared with healthy controls, OA horses exhibited significantly higher synovial concentrations of IL-1β, IL-6, TNF-α, total protein, COMP, and MMP-3. Gut microbiota analysis revealed reduced microbial diversity and significant compositional alterations in OA horses. Spearman correlation analysis demonstrated significant associations between these microbial changes and synovial inflammatory mediators as well as cartilage degradation markers. Overall, gut microbiota dysbiosis in Ili racehorses with carpal OA may be associated with synovial inflammation and abnormal cartilage metabolism via the gut–joint axis. However, the experimental scale and the number of animals will be increased as needed for further validation.

## Simple Summary

Although the gut–joint axis has been extensively investigated in osteoarthritis in humans and other animals, research on osteoarthritis in racehorses remains scarce. This study focused on racehorses from the Yili region of Xinjiang, China, selecting horses from the same area that were either affected by osteoarthritis or unaffected. Under the gut–joint axis mechanism, osteoarthritis severity was assessed using multiple methods, including lameness scores and fecal 16S rRNA, to determine gut microbiota status. Spearman correlation analysis was used to evaluate the association between gut microbiota alterations and synovial inflammation markers. The results indicate that gut microbiota dysbiosis may play an important role in synovial inflammation and cartilage metabolism in racehorse osteoarthritis through the gut–joint axis. These findings contribute to a deeper understanding of the potential role of the gut–joint axis in equine osteoarthritis and provide some reference for the early prevention and treatment of this disease in racehorses.

## 1. Introduction

The Ili horse is an excellent horse breed developed in China, originating in the Yili Kazakh Autonomous Prefecture of the Xinjiang Uygur Autonomous Region. This breed is characterized by a sturdy build, exceptional endurance, and excellent speed; it is a major domestic sport horse breed widely used in equestrian disciplines such as flat racing and endurance riding [1,2,3]. In sport horses, osteoarthritis (OA) is a common degenerative joint disease characterized by articular cartilage degeneration, synovial inflammation, and osteophyte formation. Its clinical manifestations include varying degrees of lameness, pain, and even premature retirement. According to research, 60% of lameness cases in racehorses are associated with OA [4], which seriously compromises animal welfare and causes substantial losses to the equine industry [5]. At present, the pathogenesis of OA remains unclear. Increasing evidence suggests that gut microbiota dysbiosis is associated with OA development in both humans and animals. The gut microbiota can regulate bone metabolism through the “gut–joint axis” and directly or indirectly participate in the pathological process of OA [6,7,8,9]. The “gut–joint axis” refers to the interaction between the gut and the joints through immune, endocrine, metabolic, and other pathways [10,11,12]. Collins et al. (2015) found that an increased relative abundance of *Methanotrophyta* and a decreased relative abundance of *Lactobacillus* led to elevated systemic inflammation and inflammatory mediators in the synovial fluid of a rat model [13]. Chow et al. (2024) found that the abundance of *Proteobacteria*, *Campylobacter*, and *Enterobacteriaceae* in the feces of OA horses was significantly higher [14]. An imbalance in the gut microbiota may compromise intestinal barrier integrity, allowing bacterial metabolites such as lipopolysaccharide (LPS) and peptidoglycan to enter the bloodstream, promote the release of pro-inflammatory factors, and thereby accelerate articular cartilage degeneration [15,16]. This imbalance can also lead to abnormal secretion of metabolites such as short-chain fatty acids (SCFAs) and bile acids, which affect the proliferation and differentiation of articular chondrocytes by inhibiting inflammation-related signaling pathways, thereby disrupting the balance between cartilage matrix synthesis and degradation [17,18]. Meanwhile, gut microbiota can also regulate the immune system through MIF and visfatin signaling pathways, promoting the migration of gut-derived immune cells to the joint synovium, aggravating synovial inflammation, and further advancing the progression of OA [19].

Although the relationship between gut microbiota and OA has been reported in several species, evidence in horses remains limited. This study selected Chinese Ili horses as research subjects. From the perspective of the “gut–joint axis”, it analyzed the differences in intestinal microbiota composition between racehorses with and without OA and explored its associations with synovial inflammatory mediators and cartilage degradation markers. The aim was to clarify the association between gut microbiota and OA in Ili racehorses and to provide a theoretical basis for the early diagnosis, prevention, control, and targeted intervention of this disease.

## 2. Materials and Methods

### 2.1. Experimental Animals

This study selected six Ili racehorses with arthroscopically confirmed carpus OA but without other diseases as the OA group (Group T). All horses were retired early due to pain and varying degrees of lameness caused by OA. Simultaneously, six healthy Ili horses from the same breeding area were included as the control group (Group CK). All horses were geldings; the mean ages for Group T and Group CK were 3.5 ± 0.5 years and 3.7 ± 0.4 years, respectively. The horses had similar body condition scores, were fed alfalfa, were kept at pasture, and underwent routine deworming every 4 to 5 months. All horses had a history of race participation and no history of infectious diseases or antibiotic use; no carpal trauma or septic arthritis was detected. Horses in Group T exhibited clinical signs consistent with carpal osteoarthritis, including carpal swelling, pain, lameness, and joint stiffness. Prior to inclusion in the study, all six OA-affected horses had been treated with oral Meloxicam suspension (Medaka^®^, Boehringer Ingelheim, Ingelheim, Germany), 0.6 mg/kg orally once daily for 14 days, with a washout period of at least 2 weeks before sample collection. All six horses underwent the same treatment protocol. None of the horses in the CK group had a history of treatment for carpal osteoarthritis.

All horses were from Xiyu Horse Industry Co., Ltd., Zhaosu County, Yining City, China. Synovial fluid from the carpal joint cavity and fresh feces were collected from each group using sterile apparatus for subsequent analysis of synovial fluid and gut microbiota.

### 2.2. Biochemical and Blood Routine Tests

All 12 Ili horses (6 in Group T and 6 in Group CK) underwent fasting jugular vein blood collection within 24 h before feces and joint fluid collection. Complete blood count (CBC) was performed using a fully automatic animal blood analyzer (BC-5000 Vet, Mindray, Shenzhen, China). Serum biochemical indexes were detected by a fully automatic biochemical analyzer (7020 Automatic Analyzer Hitachi, Tokyo, Japan), including liver function (ALT, AST, and ALP), renal function (UREA and Cr), total protein (TP), albumin (ALB), etc.

### 2.3. Lameness Assessment

Horses with comparable lameness scores were selected via the AAEP grading system [20]. Scores ranged from 0 (no lameness) to 5 (most severe lameness). Two veterinarians with clinical experience in horses independently performed lameness scoring, remaining blinded to the experimental group assignments during the assessment. The weighted Cohen’s kappa coefficient was used to evaluate inter-rater agreement.

### 2.4. Radiographic Examination

The radiocarpal joints of OA horses were radiographed using a digital X-ray machine (Yi’an Medical Technology Co., Ltd., Haining, China). The radiocarpal joint of the horse’s forelimb was imaged in the dorsopalmar, lateral, and oblique views to evaluate changes in joint space, bone structure, subchondral bone density, and other parameters.

Radiographic abnormalities were graded using a previously described scoring system for equine carpal osteoarthritis. Each radiographic parameter was scored on a scale of 0 to 4: 0 indicated no abnormalities; 1, minimal changes; 2, mild changes; 3, moderate changes; and 4, severe changes. The scores for joint space narrowing, osteophyte formation, and subchondral bone sclerosis were summed to obtain a total radiographic severity score for the affected carpus; higher scores indicated more severe radiographic abnormalities.

### 2.5. Arthroscopy

Horses were fasted for 8–12 h prior to the procedure. Detomidine (0.01–0.02 mg/kg) was administered intravenously for sedation. Anesthesia was induced by intravenous injection of propofol (2 mg/kg). Following endotracheal intubation, the horse was connected to an anesthesia machine, and anesthesia was maintained with inhaled isoflurane (1.2–2.0% inhaled concentration) alongside a continuous supply of oxygen.

Prior to surgery, the carpal region was routinely shaved and disinfected, and sterile drapes were applied. The affected limb was moderately weight-bearing or slightly suspended and secured. A dorsal approach to the carpus was selected, and an arthroscopic portal was established routinely medial or lateral to the common digital extensor tendons. Sterile normal saline was first injected to distend the joint cavity, after which a skin incision of approximately 5 mm was made, and the arthroscopic cannula was inserted. The dorsal structures of the carpus, including the synovium, articular cartilage surfaces (radiocarpal and middle carpal joints), synovial folds, and any loose bodies, were examined systematically. A probe was inserted through an accessory portal for palpation when necessary. Upon completion, the arthroscopic instruments were withdrawn, the intra-articular fluid was drained, and the skin incision was closed with a single suture and a sterile bandage applied.

Arthroscopic cartilage lesions were graded using a standardized 0–4 ordinal scoring system based on the OARSI recommendations for macroscopic assessment of equine osteoarthritis. Briefly, grade 0 indicated normal articular cartilage without visible lesions; grade 1 indicated mild superficial cartilage changes, such as softening, roughening, or superficial fibrillation; grade 2 indicated more pronounced fibrillation or partial-thickness cartilage erosion; grade 3 indicated extensive partial-thickness cartilage erosion or focal full-thickness cartilage defects with exposure of the calcified cartilage/subchondral surface; and grade 4 indicated severe full-thickness cartilage loss with extensive exposure of the subchondral bone.

### 2.6. Synovial Fluid Assessment

Synovial fluid was aspirated from the affected carpal joint in OA horses and from the corresponding carpal joint in control horses. The samples were placed in EDTA-containing tubes for routine analysis and stored at −80 °C for subsequent biomarker protein analysis. Synovial fluid color was scored on a scale of 1 to 5 [21] (1 = yellow; 2 = colorless; 3 = light yellow; 4 = orange; 5 = red). Transparency was graded on a scale of 1 to 3 (1 = clear; 2 = cloudy; 3 = very cloudy). Mucin content was scored on a scale of 1 to 3 (1 = good; 2 = average; 3 = poor).

### 2.7. Determination of Total Protein in Synovial Fluid Using the BCA Assay

Total protein concentrations in synovial fluid were measured using a BCA Total Protein Assay Kit (Abbkine Scientific Co., Ltd., Wuhan, China) according to the manufacturer’s instructions.

### 2.8. Enzyme-Linked Immunosorbent Assay (ELISA)

IL-1β, IL-6, and TNF-α concentrations were measured using ELISA kits (Wuhan Fine Biotech Co., Ltd., Wuhan, China). COMP and MMP-3 concentrations were measured using ELISA kits (Shanghai Fusheng Industrial Co., Ltd., Shanghai, China). The absorbance (OD value) of each sample was measured by a microplate reader, and the concentration of each biomarker was calculated against a standard curve.

### 2.9. Analysis of Faecal Microbial Diversity Composition

Frozen fecal samples were thawed on ice, and between 0.2 and 0.5 g was collected as soon as possible. The sample was added to a centrifuge tube containing the extraction lysis buffer and homogenized. The grinding instrument was a multi-sample tissue grinder from Shanghai Jingxin Company (Shanghai, China), model: Tissuelyser-48, with a grinding frequency of 60 Hz. For the pretreated samples, nucleic acids were extracted using the MagBeads FastDNA Kit for Soil (116564384) (MP Biomedicals, Santa Ana, CA, USA). The extracted DNA was then subjected to 0.8% agarose gel electrophoresis (Beijing Liuyi, Beijing, China) to determine its molecular size and quantified using Nanodrop (Thermo Scientific, Waltham, MA, USA). The highly variable V3V4 region of the bacterial 16S rRNA gene was selected for sequencing and amplified using a specific primer sequence (F: ACTCCTACGGGAGGCAGCA; R: GGACTACHVGGGTWTCTAAT). All samples were paired and sequenced on the Pasenno Gene Cloud Platform, using Pasenno’s standard bioinformatics analysis workflow, the DADA2 algorithm, and the Greengenes database for species classification and annotation.

### 2.10. Statistical Analysis

Statistical analysis was performed using SPSS 26.0 software. Measurement data are expressed as mean ± SD. Data were tested for normality using the Shapiro–Wilk test. Non-normally distributed variables are presented as median (IQR) and were analyzed using the Mann–Whitney U test. A two-sided significance level of α = 0.05 was used for statistical hypothesis testing. *p* values < 0.05 were considered statistically significant. Ordinal data were expressed as median (Q1–Q3).

To explore the potential association between inflammatory factors in synovial fluid and gut microbiota, Spearman rank correlation analysis was performed on the selected differentially expressed microbiota taxa (phylum and genus levels) and the concentrations of inflammatory factors in synovial fluid. Inflammatory factors included IL-1β, IL-6, and TNF-α, and the microbiota data were analyzed using relative abundance data. The Spearman correlation coefficient (ρ) was used to assess the monotonic correlation between inflammatory factors and bacterial abundance. To reduce the false positive rate from multiple comparisons, the Benjamini–Hochberg method was used to correct for the false discovery rate (FDR), and the corrected q-value was calculated. A difference with q < 0.05 after FDR correction was considered statistically significant, and q < 0.01 was considered highly significant. All analyses were conducted using R software (version 4.0.0). Spearman correlation coefficients and corresponding *p* values were calculated using the “psych” package, and correlation heatmaps were generated using the “pheatmap” package. In the heatmap, red indicates positive correlations and blue indicates negative correlations, with color intensity representing the strength of the correlation. Asterisks indicate significance after FDR correction (* q < 0.05, ** q < 0.01).

At the phylum level, the *Firmicutes*/*Bacteroidetes* (F/B) ratio was calculated for each horse based on the relative abundances of Firmicutes and Bacteroidetes (i.e., F/B ratio = relative abundance of *Firmicutes* ÷ relative abundance of *Bacteroidetes*). The Mann–Whitney U test was used to compare the F/B ratio between the OA group and the healthy control group. Data are presented as median and interquartile range (IQR), and a two-sided *p* < 0.05 was considered statistically significant.

## 3. Results

### 3.1. Clinical Examination and Lameness Analysis

**Figure 1:**
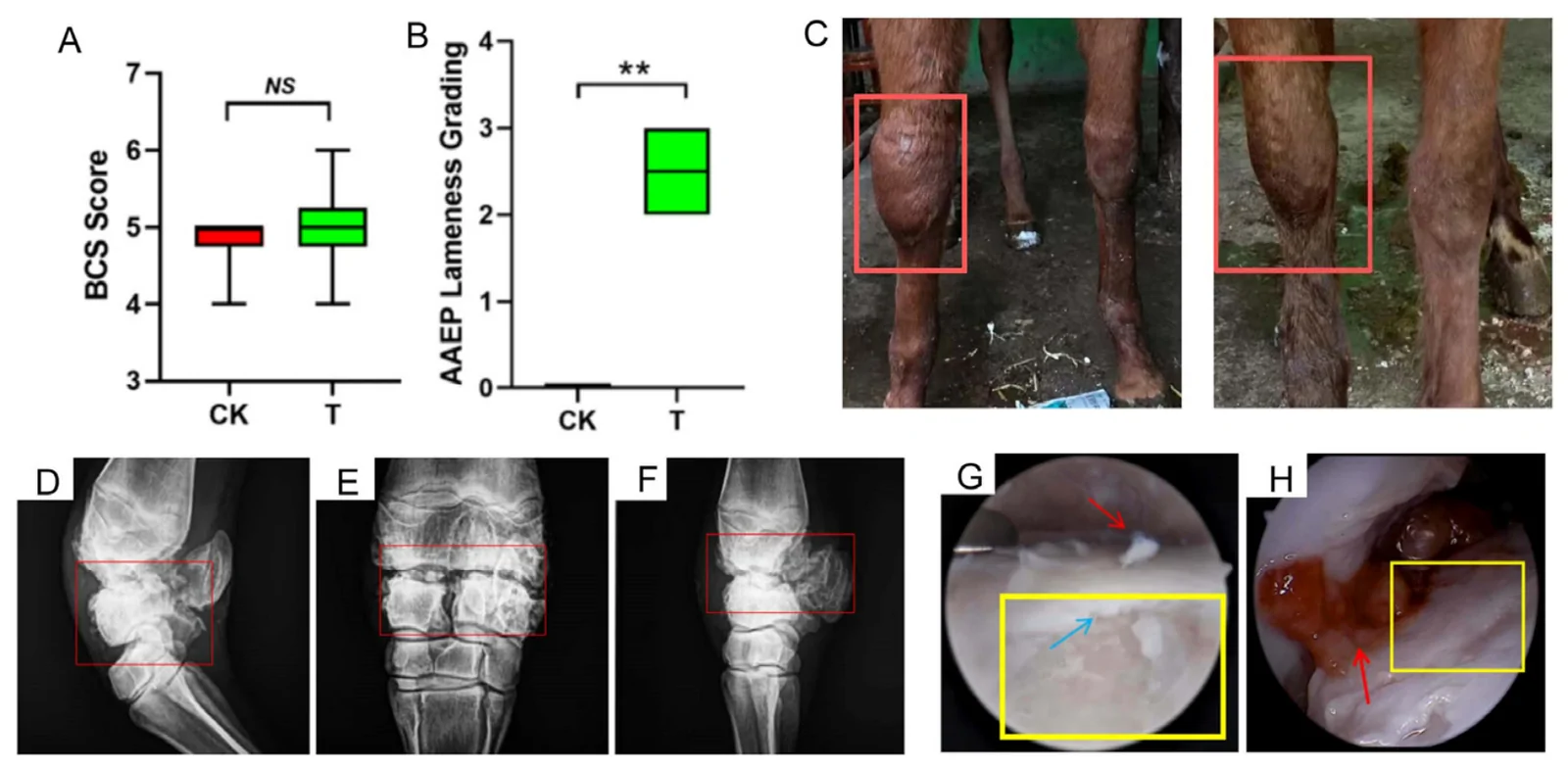
Clinical and diagnostic examinations confirmed that horses in Group T suffered from advanced carpal osteoarthritis: (**A**) There was no significant difference in BCS scores between the two groups (*p* = 0.34), NS indicates no significant difference. (**B**) The AAEP lameness score of horses in Group T was significantly higher than that in Group CK (*p* < 0.01), ** indicates a significant difference. (**C**) Representative photographs of the carpal joints of Ili racehorses from Group T; the red-boxed areas show obvious swelling in the right carpal region of the affected horses. (**D**–**F**) The carpal joint of the affected left limb shows typical radiographic features of OA: (**D**) lateral view; (**E**) dorsopalmar view; (**F**) dorsopalmar oblique view. The red boxes highlight the affected radiocarpal and midcarpal joint spaces, showing typical radiographic features of osteoarthritis, including joint space narrowing, periarticular osteophyte formation, and subchondral bone sclerosis. (**G**,**H**) Arthroscopic findings of the affected carpal joint in Group T: (**G**) The red arrows indicate free cartilage fragments, the blue arrows show the rough edges of the cartilage, and the yellow boxes show the exposed subchondral bone. (**H**) The yellow box indicates the area of cartilage degeneration, and the red arrow indicates the congested and hyperplastic synovium.

We used the BCS scoring method to assess the body condition of the horses included in the study, aiming to minimize the impact of nutritional status on the research. The body condition assessment results showed that the median BCS score for both groups was 5.0 [Q1–Q3: 5–5]. There was no significant difference in BCS scores between the two groups (*p* = 0.34) (Figure 1A). During clinical examination, the OA group horses did not exhibit obvious lameness during slow walking. However, during fast walking, the affected horses showed significant gait asymmetry, shortened stride phase, and reduced weight-bearing on the affected forelimb. According to the AAEP lameness grading system, the two evaluators showed high agreement on the AAEP lameness score (weighted Cohen’s κ = 0.886). The lameness score of Group T was significantly higher than that of the healthy control group [3.5 (3.0–4.0) vs. 0 (0–0), *p* < 0.01; Figure 1B]. The control group did not show lameness in any examination. Further local examination and palpation were performed on the OA-affected horses. Compared with the contralateral healthy joint, the OA-affected carpal joint showed significant swelling in the carpal region and increased joint circumference. During palpation, the affected horses exhibited pain-related behavioral responses, including limb withdrawal, avoidance of movement, and resistance to manipulation. Figure 1C shows representative photographs of Ili racehorses suffering from OA. Both horses exhibited marked swelling in the right carpus, consistent with the clinical signs observed in horses with osteoarthritis. Radiographic examination revealed typical degenerative changes in the affected carpus. On lateral and dorsal–palmar views, the affected joints in OA showed narrowing of the joint space, irregular joint margins, increased subchondral bone density, and periarticular osteophyte formation (Figure 1D–F). Arthroscopic examination revealed that three of the six affected joints involved the midcarpal joint, and the remaining three involved the radiocarpal joint. Typical cartilage degeneration, characterized by loss of surface smoothness, fibrosis, ulceration, and partial or full-thickness cartilage defects, is shown in Figure 1G. The synovium of the affected horse showed hyperemia, swelling, and villous hyperplasia, suggesting synovitis, as shown in Figure 1H. The results of blood tests indicated that the horses in all groups were fit for surgery and presented no anesthetic risks (Tables S1 and S2). The combined clinical, radiographic, and arthroscopic findings supported the diagnosis of advanced carpal osteoarthritis in the horses in Group T.

Arthroscopic and radiographic scoring results revealed significant articular cartilage and radiographic abnormalities in the carpal joints of all six horses in Group T. Arthroscopic scores ranged from 3 to 4. Regarding radiographic scores, the scores were 2–4 for joint space narrowing, 3–4 for osteophyte formation, and 2–4 for subchondral bone sclerosis, with a total radiographic score of 8–11. Detailed scoring results are presented in Table 1.

### 3.2. Synovial Fluid Detection and Analysis

**Figure 2:**
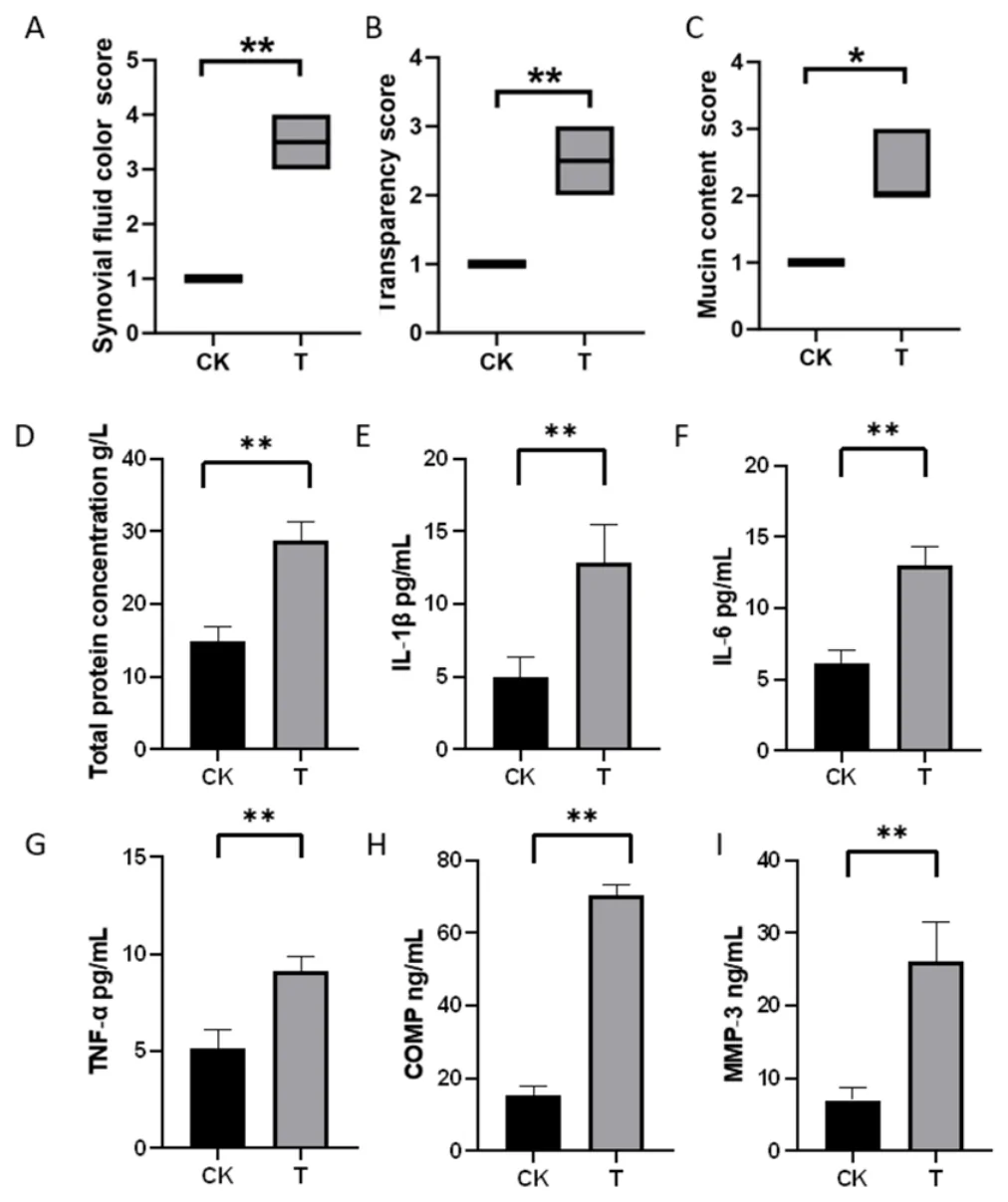
Detection of synovial fluid inflammation and cartilage metabolism indices: (**A**–**C**) Synovial fluid inflammation score (*p* < 0.05); (**D**) The total protein content in the synovial fluid of Group T was significantly higher than that of Group CK (*p* < 0.05); (**E**–**G**) The inflammatory factors in the synovial fluid of Group T were significantly higher than those in Group CK (*p* < 0.05); (**H**,**I**) The level of cartilage matrix degradation in the synovial fluid of Group T was significantly higher than that in Group CK. * indicates a significant difference; ** indicates a highly significant difference.

The synovial fluid of Group T was turbid, orange, and low viscosity, while that of Group CK was colorless, transparent, and viscous. The contents of total protein, IL-1β, IL-6, TNF-α, COMP, and MMP-3 in joint synovial fluid were detected by the BCA kit and ELISA kit, respectively, The results are shown in Figure 2. Synovial fluid analysis revealed significantly elevated concentrations of inflammatory mediators and cartilage degradation markers in OA horses. IL-1β, IL-6, and TNF-α concentrations were significantly higher in OA horses (12.82 ± 2.65, 13.03 ± 1.27, and 9.13 ± 0.77 pg/mL, respectively) than in healthy controls (4.95 ± 1.34, 6.13 ± 0.92, and 5.17 ± 0.97 pg/mL, respectively; all *p* < 0.01). Similarly, total protein, COMP, and MMP-3 concentrations were significantly increased in OA horses (28.67 ± 2.58 g/L, 70.33 ± 2.80 ng/mL, and 26.00 ± 5.48 ng/mL, respectively) compared with controls (14.83 ± 1.94 g/L, 15.50 ± 2.35 ng/mL, and 7.00 ± 1.67 ng/mL, respectively; all *p* < 0.01), indicating that there was an inflammatory reaction in the joint accompanied by the degradation of cartilage matrix.

### 3.3. Analysis of Gut Microbiota Species Composition

**Figure 3:**
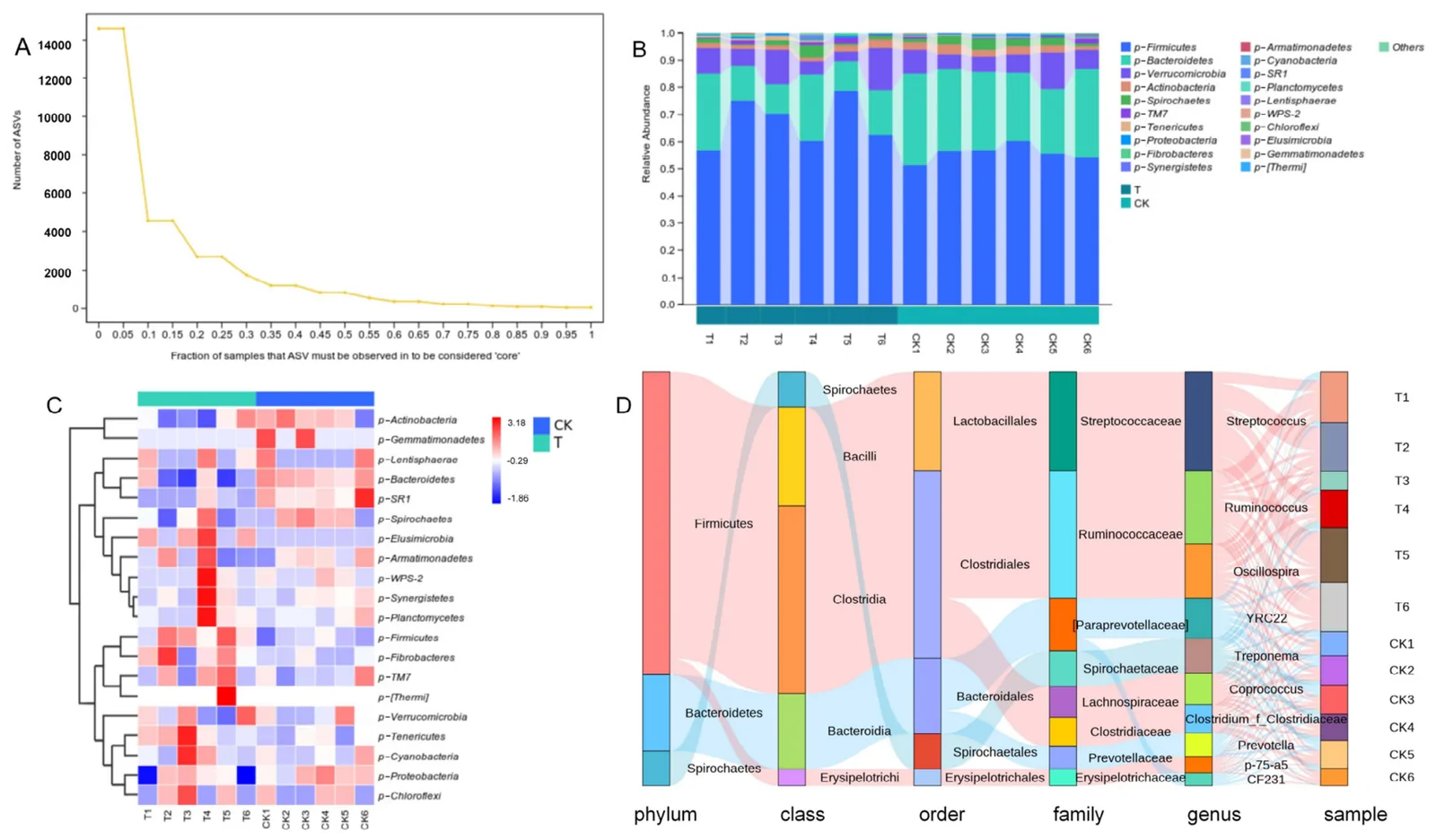
Analysis of species composition: (**A**) pan/core species analysis; (**B**) histogram of the top 20 species at the phylum level; (**C**) heatmap of the top 20 species at the phylum level; (**D**) Sankey diagram of species composition.

The pan/core species analysis demonstrated that as the frequency of ASVs in the samples increased, the corresponding number of core species gradually decreased, and the sequencing curve plateaued (Figure 3A). This indicates that the sequencing sample size was sufficient for assessing total species abundance and the number of core species. The top 20 analysis at the phylum level (Figure 3B) revealed that the relative abundance of *p_Firmicutes* was higher in Group T than in Group CK. In contrast, the relative abundances of *p_Bacteroidetes* and *p_SR1* were significantly higher in Group CK than in Group T. Compared with Group CK, the relative abundance of other phyla, such as *p_Verrucomicrobia*, *p_Actinobacteria*, and *p_Spirochaetes*, showed an increasing or decreasing trend in Group T, but the differences did not reach statistical significance (*p* > 0.05). A species composition heatmap further verified these findings (Figure 3C). Among the top 20 most abundant phyla, most showed an increasing relative abundance in Group T compared to Group CK; only the relative abundances of *p_Bacteroidetes* and *p_SR1* were higher in Group CK. The Sankey diagram revealed the flow of gut microbiota between the two groups (Figure 3D). The bacterial communities at the phylum level in each sample were mainly composed of *Firmicutes*, *Bacteroidetes*, and *Spirochetes*, with *Firmicutes* being the most dominant. At the family level, *Streptococcaceae* and *Lachnospiraceae* are the dominant bacterial groups; at the genus level, *Streptococcus*, *Ruminococcus*, *Oscillospira*, *Treponema*, and *Coprococcus* have a relatively high proportion. In Group T, the proportion of *g_Streptococcus* and *g_Ruminococcus* was relatively high, while in Group CK, the relative abundance of *g_Treponema*, *g_Coprococcus*, and some unclassified bacteria tended to be higher, suggesting that there are certain changes in the gut microbiota structure between the two groups.

At the phylum level, the relative abundance of *Firmicutes* was higher, whereas that of *Bacteroidetes* was lower in the OA group than in the healthy control group. Consistently, the F/B ratio was significantly higher in the OA group than in the healthy control group (4.85 [2.76–6.44] vs. 1.93 [1.72–2.25], respectively; Mann–Whitney U = 34.0, *p* = 0.0087).

### 3.4. Analysis of Gut Microbiota Alpha Diversity and Beta Diversity

**Figure 4:**
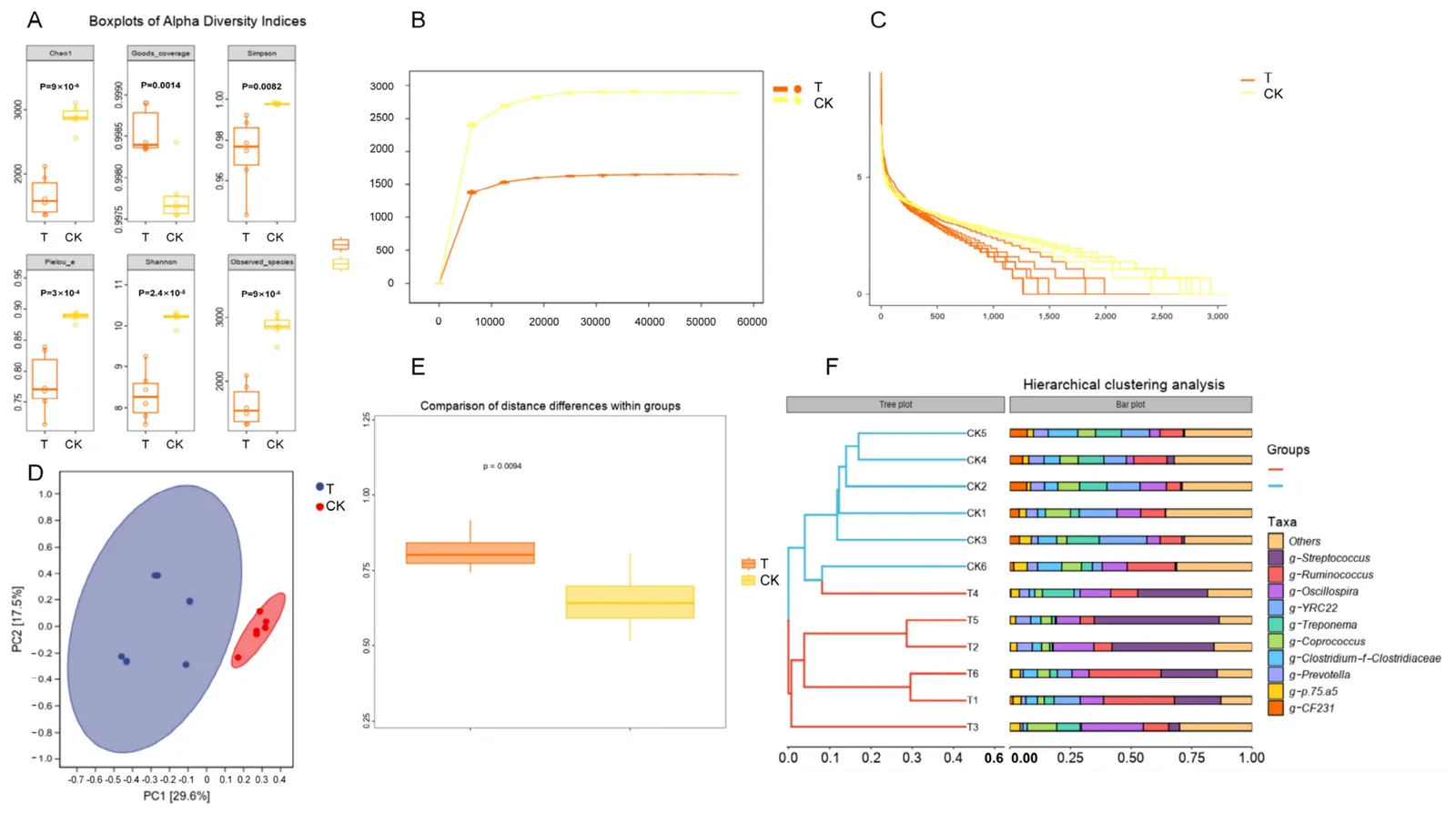
Diversity analysis of gut microbiota: (**A**) boxplot of alpha diversity index (Chao1, Good’s coverage, Simpson, Pielou’s evenness, Shannon, observed species); (**B**) sparse curve; (**C**) abundance grade curve; (**D**) PCoA analysis; (**E**) boxplot of inter-group difference analysis; (**F**) hierarchical clustering analysis diagram (sample clustering + species abundance clustering).

Alpha diversity index analysis showed that the species richness, community diversity, and species evenness of Group CK were significantly higher than those of Group T (Figure 4A). Compared with healthy controls, OA horses exhibited lower Chao1 richness (1650.11 ± 312.21 vs. 2879.64 ± 186.60), Shannon diversity (0.780 ± 0.048 vs. 0.888 ± 0.007), and observed species indices (8.31 ± 0.61 vs. 10.19 ± 0.16) (all *p* < 0.05). The abundance grade curve (Figure 4C) showed that both the number and richness of species were higher in Group CK, as was the species evenness. This further verified that the diversity of the intestinal microbiota in the healthy control group was significantly greater than that in the OA group. PCoA analysis (Figure 4D) showed that the samples from Group T and Group CK exhibited a clear separation trend in coordinate space, with the two groups mainly clustering in different regions, indicating differences in the composition of the gut microbiota between the two groups. Furthermore, Group T samples showed higher dispersion, while Group CK samples clustered more tightly, suggesting greater variation in the microbiota structure among individuals in Group T. Inter-group difference analysis (Figure 4E) revealed that the difference in bacterial flora between Group CK and Group T was extremely significant (*p* < 0.01). Hierarchical clustering analysis (Figure 4F) showed that Group T and Group CK samples basically clustered into two major branches, indicating significant differences in the composition of the two groups’ gut microbiota. Genus-level composition analysis showed that *Streptococcus*, *Ruminococcus*, and *Oscillospira* were relatively abundant in Group T, while *Treponema*, *Coprococcus*, and some unclassified bacteria accounted for a higher proportion in Group CK, further suggesting that OA has an impact on the gut microbiota structure.

### 3.5. Species Differences and Marker Analysis of Gut Microbiota

**Figure 5:**
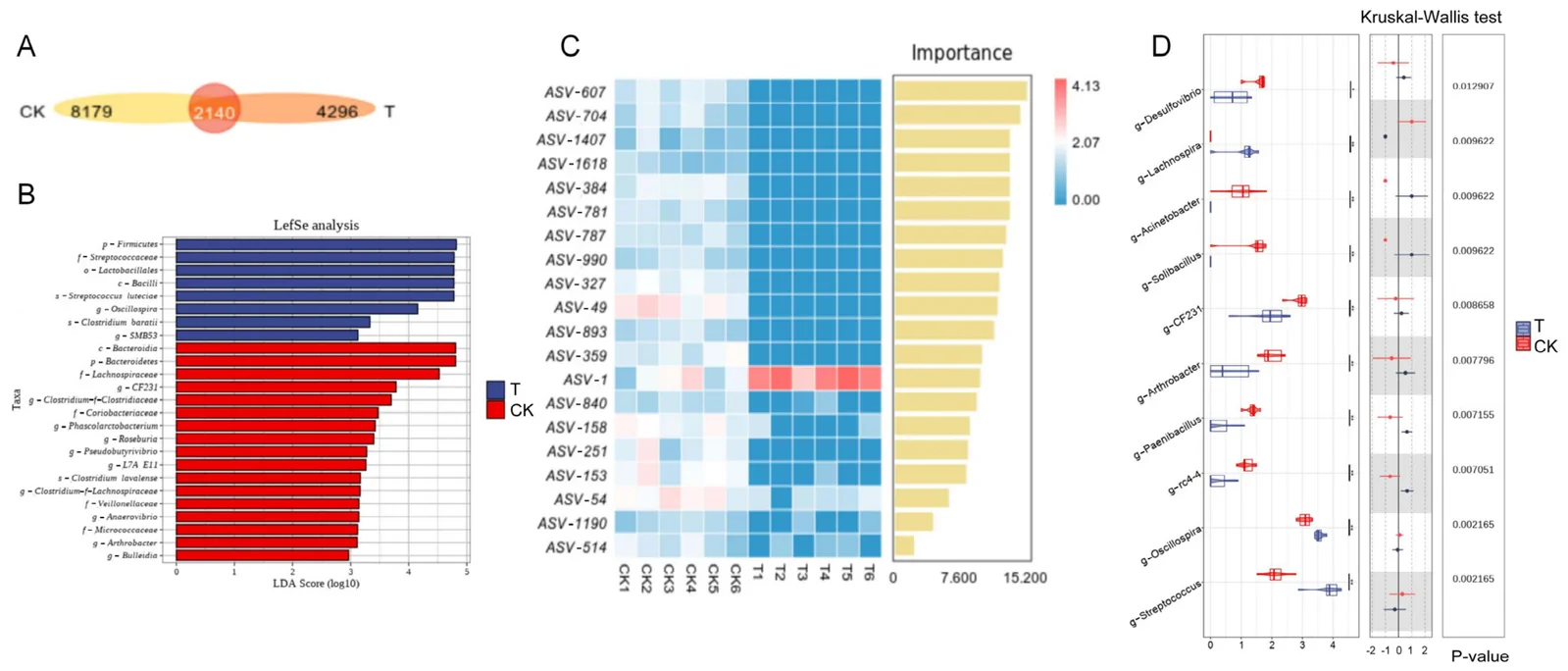
Species difference and marker analysis: (**A**) ASV/OTU Venn diagram; (**B**) LEfSe analysis cladogram; (**C**) random forest analysis species importance ranking; (**D**) volcano plot of species abundance difference test.

ASV Venn diagram analysis (Figure 5A) showed that Group CK had 8179 unique ASVs and Group T had 4296 unique ASVs, with 2140 shared between the two. This indicates significant specificity in the species composition of the gut microbiota between OA-affected and healthy horses, with the species specificity being notably higher in Group CK. Linear discriminant analysis effect size (LEfSe) was used to generate a cladogram (Figure 5B) comparing signature microbiota categories between the two groups. The results showed that *p_Firmicutes*, *g_Streptococcus*, and *g_Lactobacillales*, among others, were more abundant in Group T, whereas *p_Bacteroidetes*, *f_Lachnospiraceae*, and *g_cf231*, among others, were more abundant in Group CK.

Random forest analysis (Figure 5C) revealed that among the top 20 ASVs ranked by species importance, most were significantly more abundant in Group CK, with only the abundance of ASV_1 being higher in Group T. A species difference test (Figure 5D) confirmed that, except for *Desulfovibrio*, which showed no significant difference in abundance between the groups (*p* > 0.05), the abundance of all other highlighted bacteria was extremely significantly different between Group T and Group CK (*p* < 0.01).

### 3.6. Associations Between Differentially Abundant Gut Microbiota and Synovial Biomarkers

**Figure 6:**
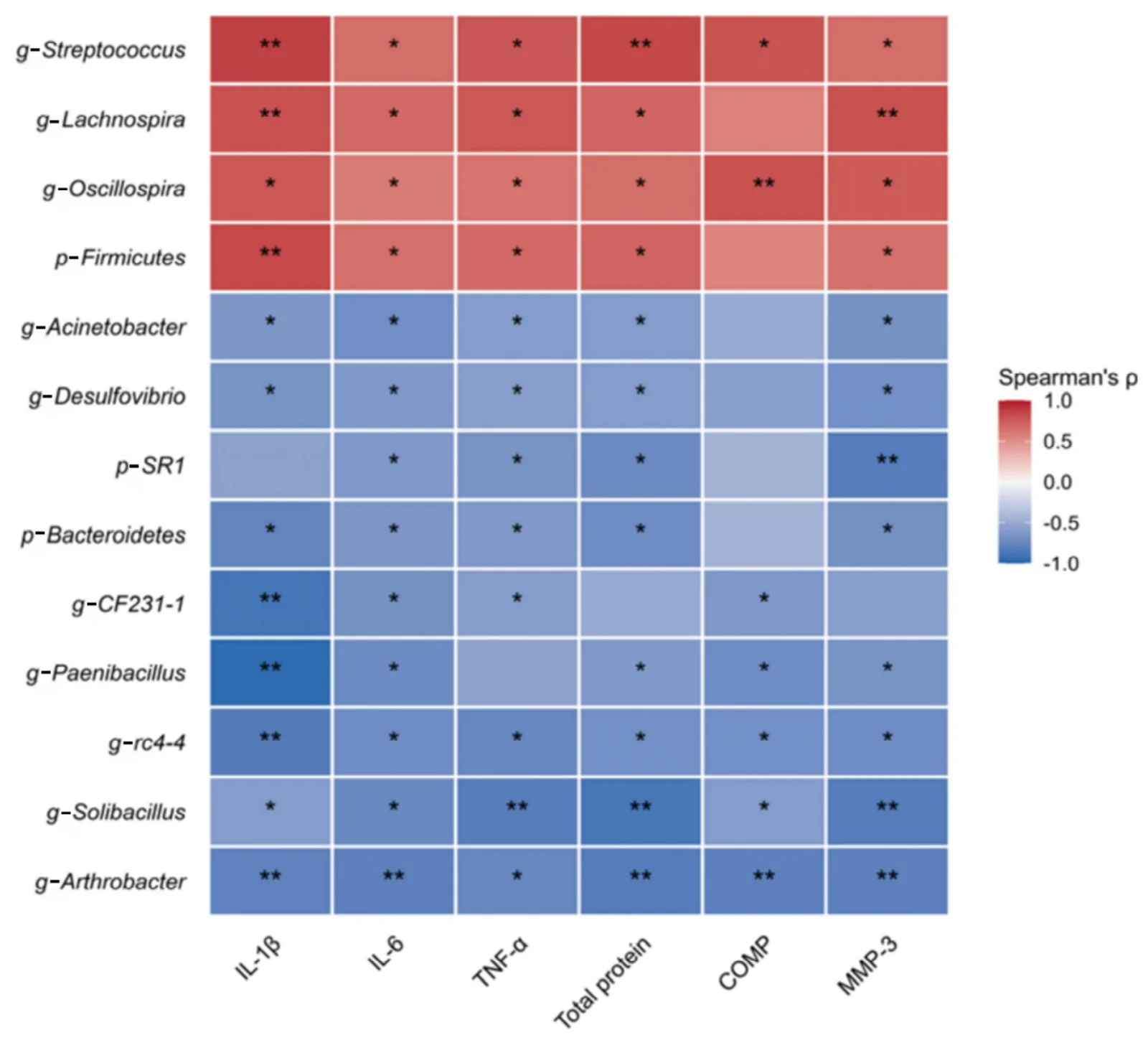
Spearman correlation heatmap showing associations between synovial fluid biomarkers and differential gut microbial taxa that differed between the two groups. Red indicates positive correlations and blue indicates negative correlations. Asterisks denote FDR-adjusted significance: * q < 0.05 and ** q < 0.01. The prefixes p and g represent phylum- and genus-level taxa, respectively.

Spearman correlation analysis was performed to further evaluate the associations between different gut microbial taxa and synovial fluid biomarkers (including IL-1β, IL-6, TNF-α, total protein, COMP, and MMP-3) in Group T and Group CK. The correlation heatmap (Figure 6) showed that several taxa enriched in OA-affected horses were positively associated with synovial inflammatory and cartilage degradation markers. At the phylum level, Firmicutes showed significant positive correlations with IL-1β, IL-6, TNF-α, total protein, and MMP-3 (FDR q < 0.05), with the association with IL-1β reaching a highly significant level (FDR q < 0.01). At the genus level, *Streptococcus*, *Lachnospira*, and *Oscillospira* displayed predominantly positive correlations with the detected synovial fluid biomarkers. *Streptococcus* and *Oscillospira* showed significant positive correlations with all six markers (FDR q < 0.05). Notably, the correlations of *Streptococcus* with IL-1β and total protein reached highly significant levels (FDR q < 0.01), while the correlation between *Oscillospira* and COMP was also highly significant (FDR q < 0.01). *Lachnospira* showed positive correlations with IL-1β, IL-6, TNF-α, total protein, and MMP-3 (FDR q < 0.05 or FDR q < 0.01).

In contrast, several taxa showed negative correlations with synovial fluid biomarkers. Bacteroidetes was negatively correlated with IL-1β, IL-6, TNF-α, total protein, and MMP-3 (FDR q < 0.05). Similarly, *Acinetobacter*, *Desulfovibrio*, *rc4-4, Paenibacillus*, *Solibacillus*, and *Arthrobacter* exhibited mainly negative correlations with inflammatory cytokines and cartilage degradation-related markers. Among these taxa, *Solibacillus* showed highly significant negative correlations with TNF-α, total protein, and MMP-3 (FDR q < 0.01), whereas *Arthrobacter* showed highly significant negative correlations with IL-1β, IL-6, total protein, COMP, and MMP-3 (FDR q < 0.01).

## 4. Discussion

In this study, horses with OA were identified by clinical examination combined with arthroscopy. The carpal joints of affected horses were enlarged and exhibited tenderness. Diagnostic imaging revealed significantly narrowed joint space and increased bone density in certain subchondral bone regions. Subsequent arthroscopic examination confirmed varying degrees of articular cartilage damage, accompanied by synovial congestion and swelling. Further analysis of synovial fluid confirmed that IL-1β, IL-6, TNF-α, COMP, and MMP-3 were significantly elevated, indicating severe intra-articular inflammation and accelerated cartilage matrix degradation.

Current evidence indicates that gut microbiota dysbiosis can allow LPS and other bacterial metabolites to enter the bloodstream through a compromised intestinal barrier, inducing systemic low-grade inflammation and directly upregulating intra-articular levels of IL-1β, IL-6, and TNF-α [22,23]. Once endotoxins and pro-inflammatory factors from the circulatory system reach the periarticular space, they can activate macrophages and fibroblast-like synovial cells [16,24,25,26], promoting the release of matrix metalloproteinases (MMPs), which degrade various protein components of the extracellular matrix (ECM) and damage articular cartilage [27]. Therefore, we performed 16S rRNA sequencing on fecal samples from both groups of racehorses. The sequencing results showed that the gut microbiota composition of OA horses was altered. Microbial richness, evenness, and diversity were significantly reduced in the OA group. PCoA analysis revealed a clear separation trend between the two groups along the coordinate axes, indicating differences in gut microbiota composition and community structure between OA horses and healthy horses. LEfSe analysis further confirmed significant differences in signature microorganisms between the two groups. The enrichment of *Streptococcal*-associated flora in Group T may be related to abnormal bile acid metabolism and disruption of host metabolic homeostasis. These changes may be associated with OA by affecting inflammatory responses and cartilage metabolism.

At the phylum level, the relative abundance of *Firmicutes* increased, while the relative abundance of *Bacteroidetes* and *SR1* decreased. The *Firmicutes*/*Bacteroidetes* (F/B) ratio is widely regarded as an important feature of many chronic inflammatory diseases and is closely associated with host metabolic disorders and enhanced inflammatory responses [28]. In this study, the F/B ratio in the OA group was higher in the OA group than in healthy horses and was accompanied by elevated levels of IL-1β, IL-6, and TNF-α, suggesting that changes in the F/B ratio may be involved in the formation of the inflammatory microenvironment in OA. At the genus level, the abundance of *Streptococcus*, *Lachnospira*, and *Oscillospira* was increased in OA horses, while the abundance of *Acinetobacter*, *Desulfovibrio*, *CF231-1*, *Paenibacillus*, *rc4-4*, *Solibacillus*, and *Arthrobacter* was decreased. Current research has confirmed that the abundance of *Streptococcus* in the gut is associated with joint diseases; the higher the abundance of *Streptococcus*, the stronger the joint pain response [29]. This phenomenon may be due to the fact that peptidoglycan, a component of the *Streptococcus* cell wall, is a potent immune activator that can induce the production of pro-inflammatory cytokines such as interferon [30]. *Lachnospira* is a core bacterial taxon that produces SCFAs, such as butyrate [31]. SCFAs play an important role in maintaining intestinal barrier integrity and exerting systemic anti-inflammatory effects by inducing Treg cells [32], suggesting that the gut may have stronger anti-inflammatory capacity when such bacteria are dominant.

Further correlation analysis revealed significant associations between OA-related differentially expressed microbiota and synovial inflammatory factors and cartilage degradation markers. Specifically, *p_Firmicutes* and genera including *Streptococcus*, *Lachnospira*, and *Oscillospira* were significantly positively correlated with synovial inflammatory mediators and cartilage degradation markers, with *p_Firmicutes* showing the strongest association with IL-1β and *g_Oscillospira* exhibiting a particularly strong correlation with COMP. In contrast, the *p_SR1*, *g_Solibacillus*, and *g_Arthrobacter* were negatively correlated with most synovial biomarkers, suggesting potential protective roles. Furthermore, *Streptococcus* showed a significant positive correlation with all three inflammatory factors, with the strongest correlation observed with IL-1β. Interestingly, *g_Lachnospira* and *g_Oscillospira* were positively correlated with inflammatory factors and cartilage degradation markers in this study. Notably, *g_Lachnospira* showed significant positive associations with IL-1β, IL-6, TNF-α, and MMP-3, whereas *g_Oscillospira* was not only positively correlated with inflammatory cytokines but also particularly strongly associated with COMP. These findings appear inconsistent with the conventional view that both genera belong to SCFA-producing bacteria that contribute to intestinal homeostasis and immune regulation [33]. This discrepancy may reflect compensatory proliferation of certain related bacteria under chronic inflammation due to disruption of the microbial ecosystem.

However, given the relatively small sample size of this study, future research involving larger cohorts and a wider range of types is warranted. Furthermore, factors such as pain, stress, and reduced physical activity may also influence the composition of the gut microbiota. Consequently, the findings of this study should be interpreted as evidence of association rather than causation. Future studies building upon this work will further investigate the molecular and pathological mechanisms linking horse OA and the gut.

## 5. Conclusions

The richness, diversity, and evenness of the gut microbiota were reduced in OA horses, and the composition and community structure of the microbiota were significantly altered. These changes were mainly characterized by enrichment of taxa such as *Firmicutes*, *Streptococcus*, *Lachnospira*, and *Oscillospira*, while the abundance of *Bacteroidetes* and several other microbial groups decreased. Correlation analysis showed that some differentially expressed OA-related microbiota were significantly correlated with synovial inflammatory factors and cartilage degradation markers, suggesting that gut microbiota dysbiosis may be related to synovial inflammation and abnormal cartilage metabolism in equine OA. Given the relatively small sample size of this study, future research should increase the number of animals and conduct further experiments to validate these findings.

## Figures and Tables

**Table 1:** Arthroscopic cartilage scores and radiographic scores of the carpal joint in Group T.

		Arthroscopic Score	Joint Space Narrowing	Osteophyte Formation	Subchondral Bone Sclerosis	Total Radiomics Score
T1	midcarpal	4	3	4	4	11
T2	midcarpal	3	3	3	2	8
T3	radiocarpal	3	2	3	3	8
T4	midcarpal	4	3	4	3	10
T5	radiocarpal	4	4	4	2	10
T6	radiocarpal	4	4	3	4	11

## Data Availability

The original contributions presented in this study are included in the article. Further inquiries can be directed to the corresponding author.

## Supplementary Materials

The following supporting information can be downloaded at https://www.mdpi.com/article/10.3390/ani16203299/s1, Table S1: Results of hematological examinations in healthy and OA horses; Table S2: Results of biochemical routine examinations in healthy and OA horses.

## Abbreviations

The following abbreviations are used in this manuscript:

OA	Osteoarthritis
BCS	Body Condition Score
AAWP	American Association of Equine Practitioners
NSAIDs	Non-Steroidal Anti-Inflammatory Drugs
IV	Intravenous
OD	Optical Density
BCA	Bicinchoninic Acid
CBC	Complete Blood Count
ALT	Alanine Aminotransferase
AST	Aspartate Aminotransferase
ALP	Alkaline Phosphatase
Cr	Creatinine
TP	Total Protein
ALB	Albumin
IL-1β	Interleukin-1 beta
IL-6	Interleukin-6
TNF-α	Tumor Necrosis Factor-alpha
COMP	Cartilage Oligomeric Matrix Protein
MMP-3	Matrix Metalloproteinase-3
LPS	Lipopolysaccharide
SCFAs	Short-Chain Fatty Acids
ECM	Extracellular Matrix
ASV	Amplicon Sequence Variant
LEfSe	Linear Discriminant Analysis Effect Size
PCoA	Principal Coordinates Analysis
