# Supplementary material for: Association Between Gut Microbiota Dysbiosis and Synovial Inflammation in Carpal Osteoarthritis of Ili Racehorses: A Preliminary Study Integrating Imaging, Synovial Fluid Analysis, and Fecal 16S rRNA Sequencing
Source: Animals (Basel). 2026 Oct 20;16(20):3299. doi: 10.3390/ani16203299 (PMC13700779; doi:10.3390/ani16203299)
Supplement: Supplementary file 1 [file animals-16-03299-s001.zip › animals-4582527-supplementary.pdf]

Prior to arthroscopic surgery, complete blood counts and biochemical analyses were performed on horses in all groups to assess their baseline health status and ensure their physical condition was suitable for the procedure. The results indicated that the horses in all groups were fit for surgery and presented no anesthetic risks (Table S1 and S2).

Table S1 Results of hematological examinations in healthy and OA horses.

Index	Unit	CK group (Healthy control group)	T group (Osteoarthritis group)	Reference range
White Blood Cell (WBC)	$\times 10^9$/L	8.24 ± 1.56	8.67 ± 1.82	5.00 – 12.00
Neutrophil (NEU)	$\times 10^9$/L	4.56 ± 1.20	5.78 ± 1.35	2.18 – 6.96
Lymphocyte (LYM)	$\times 10^9$/L	3.15 ± 0.68	3.28 ± 0.74	1.32 – 5.86
Monocyte (MONO)	$\times 10^9$/L	0.42 ± 0.12	0.45 ± 0.14	0.05 – 0.92
Eosinophil (EOS)	$\times 10^9$/L	0.36 ± 0.11	0.26 ± 0.17	0.01 – 1.00
Basophil (BAS)	$\times 10^9$/L	0.02 ± 0.01	0.04 ± 0.02	0.00 – 0.12
Red Blood Cell (RBC)	$\times 10^{12}$/L	8.15 ± 1.08	7.92 ± 1.15	5.30 – 10.50
Hemoglobin (HGB)	g/L	135.67 ± 15.42	131.83 ± 16.28	100.00 – 170.00
Hematocrit (HCT)	%	38.52 ± 4.36	37.64 ± 4.58	30.00 – 49.00
Mean Corpuscular Volume (MCV)	fL	47.32 ± 3.54	47.56 ± 3.68	37.00 – 59.00
Mean Corpuscular Hemoglobin (MCH)	pg	16.75 ± 1.32	16.83 ± 1.40	13.50 – 19.50
Mean Corpuscular Hemoglobin Concentration (MCHC)	g/L	352.33 ± 14.68	349.17 ± 15.22	300.00 – 380.00
Platelet (PLT)	$\times 10^9$/L	186.50 ± 32.45	192.33 ± 35.78	90.00 – 360.00
Mean Platelet Volume (MPV)	fL	6.77 ± 0.33	6.89 ± 0.24	5.50 – 8.00

Data are presented as mean ± SD.CK group (n = 6); T group (n = 6).

Table S2 Results of biochemical routine examinations in healthy and OA horses.

Index	Unit	CK group (Healthy control group)	T group (Osteoarthritis group)	Reference range
Alanine Aminotransferase (ALT)	U/L	6.28 ± 0.52	8.33 ± 1.16	5.00 – 50.00
Aspartate Aminotransferase (AST)	U/L	239.05 ± 10.03	277.30 ± 20.51	160.00 – 412.00
Alkaline Phosphatase (ALP)	U/L	225.10 ± 44.30	243.21 ± 43.21	138.00 – 251.00
UREA	mmol/L	5.88 ± 0.35	5.96 ± 0.44	4.00 – 8.00
Creatinine (Cr)	μmol/L	86.96 ± 18.20	99.24 ± 30.62	80.00 – 140.00
Total Protein (TP)	g/L	68.33 ± 1.27	73.02 ± 3.66	56.00 – 79.00
Albumin (ALB)	g/L	29.90 ± 0.66	30.19 ± 2.03	19.00 – 32.00
Total Calcium (Ca)	mmol/L	3.02 ± 0.21	3.12 ± 0.13	2.78 – 3.32

Data are presented as mean ± SD.CK group (n = 6); T group (n = 6).
